# Pre-filter that incorporates the noise model

**DOI:** 10.1186/s42492-020-00051-z

**Published:** 2020-05-22

**Authors:** Gengsheng L. Zeng

**Affiliations:** 1 Department of Engineering, Utah Valley University, 800 West University Parkway, Orem, UT 84058 USA; 2 Department of Radiology and Imaging Sciences, University of Utah, 729 Arapeen Drive, Salt Lake City, UT 84108 USA

**Keywords:** Low-dose computed tomography, Denoising, Linear filter, Nonstationary filter

## Abstract

A linear denoising filter is usually of lowpass type, and the main parameter in a lowpass filter is the cutoff frequency. The lowpass filters are normally shift invariant and can be implemented as convolution in the spatial domain or as multiplication in the Fourier domain. This paper presents a linear filter that is not characterized by its cutoff frequency but is characterized by the noise model. An example of such a linear filter is presented for low-dose X-ray computed tomography (CT).

## Introduction

Nowadays, the iterative image reconstruction algorithms have become more popular than the analytic filtered backprojection (FBP) algorithm [1–5]. The main motivation of using an iterative algorithm is that it is much easier to incorporate the noise model into the iterative algorithm than into an analytic algorithm such as FBP [6, 7]. Many noise filtering techniques are available for the FBP algorithm [8–11]. In ref. [9], a non-linear adaptive filter was proposed, in which a threshold value was assigned to determine whether the measurements were required to be filtered.

In refs. [11, 12], a linear FBP algorithm was proposed to mimic the iterative Landweber algorithm [13] and to incorporate the projection noise in a window function in the Fourier domain. The ray-by-ray noise-weighting scheme is spatially variant. Its implementation was to decompose the spatially variant filter into 10 spatially invariant filters. The 10 versions of the filtered measurements were then combined into one and used in the backprojector.

Instead of mimicking an iterative algorithm to filter the noise, this paper takes a different approach and develops a convolution lookalike linear filter for the projection measurements. The filter being designed is shift variant and is based on the noise model. We treat each measurement as a random variable. By noise model, we mean the relationship between the measurement value and its standard deviation. For emission tomography measurements, the mean value is approximated by its measurement. The Poisson noise model is that the mean value and the variance are the same. For the transmission tomographic measurements, the logarithm has been taken. The variance of the line-integral measurement is proportional to the exponential function of the line-integral measurement.

## Method

This section develops a nonstationary convolution lookalike filter for the line-integral projection measurement data. Let *p* be the unfiltered line-integral projection measurement and *h* be the spatial-domain filter kernel. In other words, *p* is the logarithm of the detected photon current from a computed tomography (CT) scanner bin at an angle. If *h* is a shift-invariant convolution kernel, the conventional linear shift-invariant filtering procedure can be expressed as a convolution integral below

1 $$ q\left(t,\uptheta \right)=\iint h\left(\hat{t}-t,\hat{\theta}-\uptheta \right)p\left(\hat{t},\hat{\theta}\right)\ d\hat{t}d\hat{\theta}\kern2.25em $$

where *q*(*t*,*θ*) is the filtered projection. In Eq. (1), the kernel *h* is shift-invariant. In other words, the shift-invariant filter blurs the projections *p*(*t*,*θ*) with the same kernel *h* everywhere. If the kernel *h* varies from location to location, Eq. (1) can be modified to

2 $$ q\left(t,\uptheta \right)=\iint h\left(\hat{t},\hat{\theta};t,\uptheta \right)p\left(\hat{t},\hat{\theta}\right)\ d\hat{t}d\hat{\theta}\kern1.75em $$

Equation (2) is no longer in the form of convolution. However, the computational complexity of Eq. (2) is almost the same as the complexity of Eq. (1), except that in Eq. (2) the kernel *h* must be evaluated differently for different locations (*t*,*θ*). In this paper, we assume the filter kernel *h* to be a two dimensional (2D) Gaussian function with a ‘standard deviation’ σ_*h*_(*t*,*θ*). Our general strategy is to relate this σ_*h*_(*t*,*θ*) with the noise standard deviation, σ_*p*_(*t*,*θ*), of the noisy measurement *p*(*t*,*θ*).

For emission measurements, the projection *p*(*t*,*θ*) can be used to approximate the measurement noise variance according the Poisson distribution. Thus, ${\sigma}_p\left(t,\theta \right)\approx \sqrt{p\left(t,\theta \right)}$.

For Transmission measurements, the noise variance of the post-log measurement *p*(*t*,*θ*) can be approximated as the exponential function of the line-integral *p*(*t*,*θ*). Thus, ${\sigma}_p\left(t,\theta \right)\approx \sqrt{\exp \left(p\left(t,\theta \right)\right)}$.

Our general strategy is to use a large kernel size of *h* if the corresponding σ_*p*_(*t*,*θ*) value is large and to use a small kernel size if the corresponding σ_*p*_(*t*,*θ*) value is small. The kernel size σ_*h*_(*t*,*θ*) is thus a monotonic function of σ_*p*_(*t*,*θ*). Here we propose this monotonic function to have the form of

3 $$ {\sigma}_h=a\times {\sigma}_p^b+c\kern0.75em $$

For transmission tomography, Eq. (3) becomes

4 $$ {\sigma}_h=a\times {\left(\sqrt{e^{p\left(t,\theta \right)}}\right)}^b+c\kern1.25em $$

After combining the square-root and parameter *b* into a single parameter *b*, Eq. (4) becomes

5 $$ {\sigma}_h=a\times {e}^{b\times p\left(t,\theta \right)}+c\kern1em $$

with user defined parameters *a*, *b*, and *c*.

As a special case of *a* = 0, *h* has a constant σ_*h*_, the filter is then shift-invariant, and the convolution lookalike expression Eq. (2) reduces to the true convolution Eq. (1).

## Results

To illustrate the feasibility of the proposed linear nonstationary filter, a cadaver torso was scanned using an X-ray CT scanner with a low-dose setting. The projections were first filtered by the proposed filter using Eq. (2) and then the conventional FBP algorithm was used to reconstruct the final image.

The cadaver data was collected with a diagnostic scanner (Aquilion ONE™, Toshiba America Medical Systems, Tustin, CA, USA; raw data courtesy of Leiden University Medical Center). The imaging geometry was cone-beam, the X-ray source trajectory was a circle of radius 600 mm. The detector had 320 rows, the row-height was 0.5 mm, each row had 896 channels, and the fan angle was 49.2°. A low-dose noisy scan was carried out. The tube voltage was 120 kV and current was 60 mA. There were 1200 views uniformly sampled over 360°. Three patient slices were selected to test our proposed spatially variant filter.

**Fig. 1:**
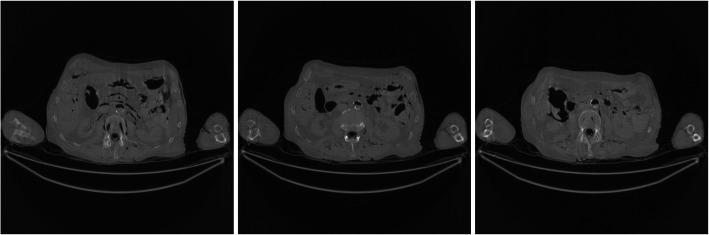
FBP reconstruction of three image slices using the regular-dose data without pre-filtering. These images are the gold standard images for other images to compare with. Left to Right: Slice 1, Slice 32, and Slice 64

Figure 1 shows the FBP reconstruction of three image slices from the low-dose CT data. It is observed that some horizontal streaking artifacts pass through both arms and the torso. The X-rays passing through both arms suffer from photon starvation and create large noise.

**Fig. 2:**
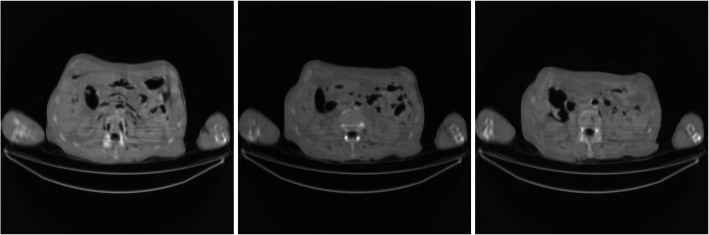
FBP reconstruction of three image slices using a nonstationary Gaussian pre-filter *h* whose *σ*_*h*_ is defined in Eq. (5) with *a* = 2.5, *b* = 4, and *c* = −2.49975. Left to Right: Slice 1, Slice 32, and Slice 64

**Fig. 3:**
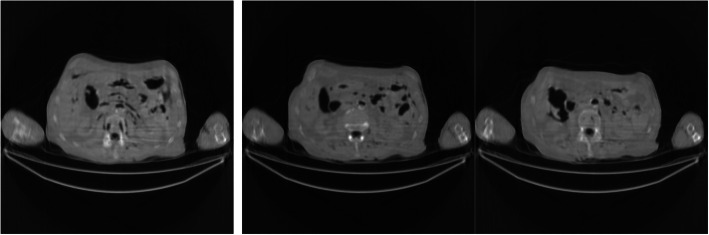
FBP reconstruction of three image slices using a nonstationary Gaussian pre-filter *h* whose *σ*_*h*_ is defined in Eq. (5) with *a* = 2.5, *b* = 3, and *c* = − 2.49975. Left to Right: Slice 1, Slice 32, and Slice 64

**Fig. 4:**
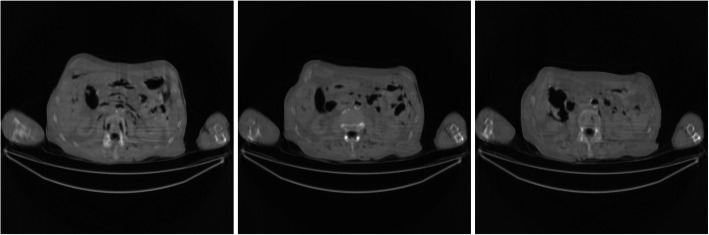
FBP reconstruction of three image slices using a nonstationary Gaussian pre-filter *h* whose *σ*_*h*_ is defined in Eq. (5) with *a* = 2.5, *b* = 2, and *c* = − 2.49975. Left to Right: Slice 1, Slice 32, and Slice 64

**Fig. 5:**
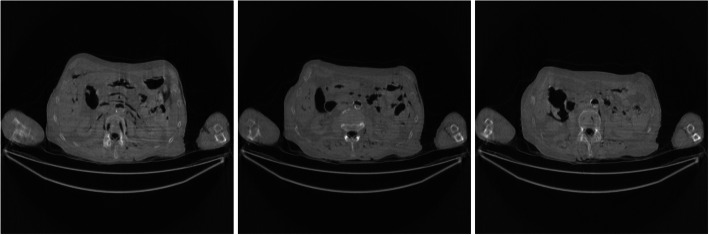
FBP reconstruction of three image slices using a nonstationary Gaussian pre-filter *h* whose *σ*_*h*_ is defined in Eq. (5) with *a* = 2.5, *b* = 1, and *c* = − 2.49975. Left to Right: Slice 1, Slice 32, and Slice 64

**Fig. 6:**
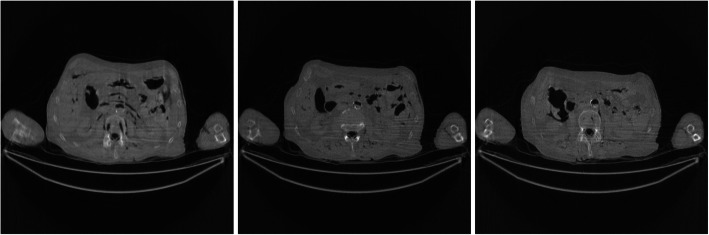
FBP reconstruction of three image slices using a nonstationary Gaussian pre-filter *h* whose *σ*_*h*_ is defined in Eq. (5) with *a* = 2.5, *b* = 0.5, and *c* = − 2.49975. Left to Right: Slice 1, Slice 32, and Slice 64

Figures 2, 3, 4, 5, 6 show the FBP reconstructions of three image slices from the pre-filtered low-dose CT data. The pre-filters were nonstationary, the associated kernel *h* was a Gaussian function, whose ‘standard deviation’ value *σ*_*h*_ was defined in Eq. (5). They shared the same values of *a* = 2.5 and *c* = − 2.49975. The *b* value was different, with *b* = 4, 3, 2, 1, and 0.5, respectively. It seems that a large *b* value helps removing the streaking artifacts.

**Fig. 7:**
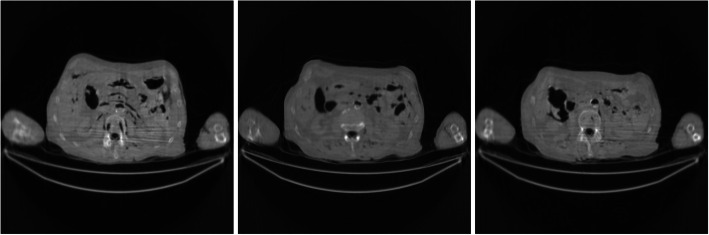
FBP reconstruction of three image slices using a stationary Gaussian pre-filter *h* whose *σ*_*h*_ is defined in Eq. (5) with *a* = 0 and *c* = 2. Left to Right: Slice 1, Slice 32, and Slice 64

**Fig. 8:**
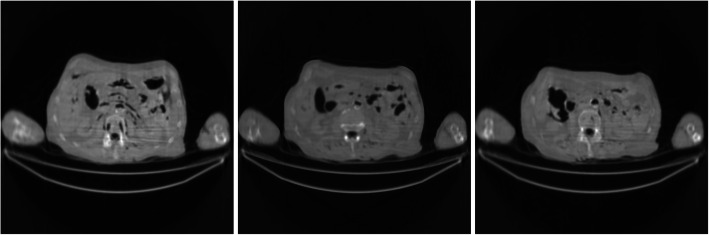
FBP reconstruction of three image slices using a stationary Gaussian pre-filter *h* whose *σ*_*h*_ is defined in Eq. (5) with *a* = 0 and *c* = 4. Left to Right: Slice 1, Slice 32, and Slice 64

Figures 7 and 8 show the FBP reconstructions from the pre-filtered low-dose CT data. This time, the pre-filters were stationary, that is, the kernel *h* was shift-invariant by setting *a* = 0. The parameter *c* controls the degree of smoothing during filtering. In Figs. 7 and 8, *c* = 2 and 4, respectively. For a stationary filter, the entire image is smoothed at the same amount. It is observed that the stationary filters are not effective in removing streaking artifacts. The image is already blurry, while the streaking artifacts are still there.

**Fig. 9:**
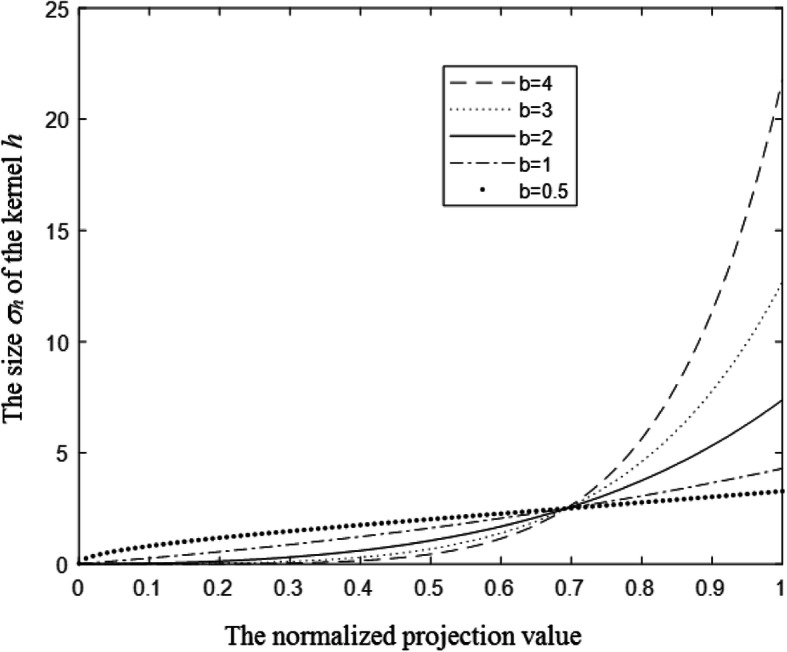
The functional relationship Eq. (5) of 5 cases as in Figs. 2 to 5, 10, respectively. The horizontal axis represents the normalized projection value, and the vertical axis represents the size *σ*_*h*_ of the kernel *h*

**Fig. 10:**
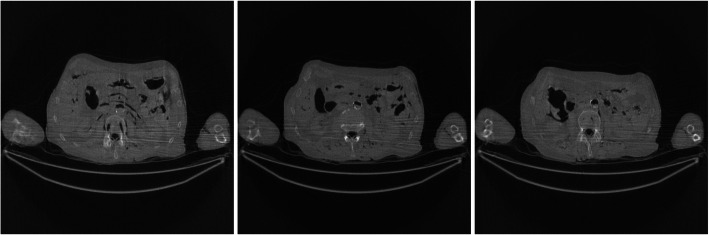
FBP reconstruction of three image slices using the low-dose data without pre-filtering. Left to Right: Slice 1, Slice 32, and Slice 64

Figure 9 shows 5 curves according to the relationship Eq. (5). The 5 curves correspond to the 5 cases with different *b* values as shown in Figs. 2, 3, 4, 5, 10, respectively. The horizontal axis represents the normalized projection value, and the vertical axis represents the size *σ*_*h*_ of the kernel *h*.

When the parameter *b* is small such as 0.5, the kernel size *σ*_*h*_ increases gently with the projection *p*(*t*,*θ*). When the parameter *b* is large such as 4, the kernel size *σ*_*h*_ increases dramatically with the projection *p*(*t*,*θ*). Intuitively speaking, the streaking artifacts are caused by a few projections with the largest values, whose rays pass through both arms. Using a large parameter *b* (e.g., *b* = 2) effectively separates the projections with large values from other projections. Only those large projection values are smoothed, while other smaller projection values are almost unchanged. The overall image resolution is unchanged.

The effectiveness of artifact removal of the proposed spatially variant filter can by assessed by visual inspection of the reconstructed images. It can also be evaluated by a numerical figure-of-merit, called artifact index, which is empirically developed and explained as follows.

The artifact index compares a low-dose image against its corresponding regular-dose image using the following steps. Step 1: Obtain a pair the reconstructed images, one with regular-dose data and the other one with low-dose data. Step 2: Extract the edges (i.e., features) of these images using the Canny method. We used Matlab’s built-in function edge (Image, ‘Canny’) to accomplish this step. Step 3: Evaluate the difference image of these two edge images. Step 4: Evaluate the element-by-element square of the error image. Step 5: Small isolated dot-like features are removed by using Matlab’s built-in function bwareaopen (Image, 70). Step 6: Find the artifact index as the total sum of the image values in processed image from Step 5. The results of the artifact index values for reconstructed images are summarized in Table 1. A smaller artifact index may imply fewer artifacts being detected from the low-dose image.

**Table 1:** Artifact index study for some reconstructed images

Cases	Filter parameters			Noise filter (Low-dose data, FBP reconstruction)	Artifact indices		
	a	b	c		Slice 1	Slice 32	Slice 64
1	NA	NA	NA	No filter	10,620	5030	8154
2	2.5	4	−2.49975	Proposed filter	6285	4176	4718
3	2.5	3	−2.49975	Proposed filter	5046	3756	3676
4	2.5	2	−2.49975	Proposed filter	3787	3597	3434
5	2.5	1	−2.49975	Proposed filter	3991	2524	3524
6	2.5	0.5	−2.49975	Proposed filter	7306	4495	5909
7	0	NA	2	Lowpass filter	4930	3653	4103
8	0	NA	4	Lowpass filter	7698	5745	6959

## Conclusions

The proposed pre-filter is designed for the FBP algorithm, aiming to reduce the photon starvation streaking artifacts. This is a linear filter as suggested by Eq. (2). It has a convolution lookalike form, with similar computational complexity as convolution.

The most important property of the proposed filter is its nonstationary characteristic, which allows the filter to smooth the data with specified range of values. As a result, some streaking artifacts can be reduced. Stationary filters do not have this ability.

The proposed spatially variant filter is a lowpass filter with a small kernel span. The low frequency components are almost not affected. However, at the same time of suppressing high frequency artifacts at certain views, some high frequency contrasts are suppressed as well. The low frequency image quantitation is almost not affected, but some high frequency details may get lost.

A mathematical analysis would conclude that the optimal value of parameter *b* in Eq. (5) should be 0.5 because it matches the noise model of the transmission tomography. However, computer simulations reveal that this is not the case and the *b* value should be larger than 0.5 to be effective. We still do not understand why it is more effective than the ‘correct’ value of *b* = 0.5. A previous paper published in 2016 [14] presented many examples to illustrate this un-explained phenomenon and concluded that the optimal parameter depends on the object and the image contrast. Therefore, there is no universal optimal parameter. The so-called ‘correct’ weighting function is sub-optimal.

A low-dose CT study is used to test the feasibility of the proposed pre-filter. The relationship between the kernel size and the noise variance is not uniquely determined. The model (5) is only one functional form that can be utilized. The user is encouraged to adopt other forms to label the undesired projection values to be smoothed out.

## Data Availability

Not applicable.

## Abbreviations

CT: Computed tomography
FBP: Filtered backprojection
2D: Two dimensional
